# Supplementary material for: Use of standardised patients to assess tuberculosis case management by private pharmacies in Patna, India: A repeat cross-sectional study
Source: PLOS Glob Public Health. 2023 May 26;3(5):e0001898. doi: 10.1371/journal.pgph.0001898 (PMC10218738; doi:10.1371/journal.pgph.0001898)
Supplement: Attachment — Submitted filename: Response to Reviewers.docx [file pgph.0001898.s001.docx]

PGPH-D-23-00255

Dr Dan Kajungu, PhD
Academic Editor
PLOS Global Public Health

Dear Dr Kanjungu

Thank you for the opportunity to revise and resubmit an improved manuscript. As you can see below, we have addressed all the comments from both reviewers. We hope you find this version acceptable.

Best regards

Prof Madhukar Pai, MD, PhD

Canada Research Chair in Epidemiology & Global Health

Associate Director, McGill International TB Centre

McGill School of Population and Global Health

McGill University

2001 McGill College Avenue, Suite 1200

Montreal QC H3A 1G1

Email: [madhukar.pai@mcgill.ca](mailto:madhukar.pai@mcgill.ca)

**Response to Reviewers**

*Thank you to both reviewers for providing such interesting and helpful comments. Our responses are italicized after each corresponding. comment.*

Reviewers' comments:

Reviewer's Responses to Questions

**Comments to the Author**

1. Does this manuscript meet PLOS Global Public Health’s [publication criteria](https://can01.safelinks.protection.outlook.com/?url=https%3A%2F%2Fjournals.plos.org%2Fglobalpublichealth%2Fs%2Fcriteria-for-publication&data=05%7C01%7Canita.svadzian%40mail.mcgill.ca%7C9ecd4c3930ad41c7bada08db2aa55fb8%7Ccd31967152e74a68afa9fcf8f89f09ea%7C0%7C0%7C638150661979815228%7CUnknown%7CTWFpbGZsb3d8eyJWIjoiMC4wLjAwMDAiLCJQIjoiV2luMzIiLCJBTiI6Ik1haWwiLCJXVCI6Mn0%3D%7C3000%7C%7C%7C&sdata=pJP7BdSFxppkbMzhxa8TDu2JcZTj5%2F9hCBfWVVgDrlY%3D&reserved=0)? Is the manuscript technically sound, and do the data support the conclusions? The manuscript must describe methodologically and ethically rigorous research with conclusions that are appropriately drawn based on the data presented.

Reviewer #1: Yes

Reviewer #2: Yes

2. Has the statistical analysis been performed appropriately and rigorously?

Reviewer #1: Yes

Reviewer #2: Yes

3. Have the authors made all data underlying the findings in their manuscript fully available (please refer to the Data Availability Statement at the start of the manuscript PDF file)?

The [PLOS Data policy](https://can01.safelinks.protection.outlook.com/?url=https%3A%2F%2Fjournals.plos.org%2Fglobalpublichealth%2Fs%2Fdata-availability&data=05%7C01%7Canita.svadzian%40mail.mcgill.ca%7C9ecd4c3930ad41c7bada08db2aa55fb8%7Ccd31967152e74a68afa9fcf8f89f09ea%7C0%7C0%7C638150661979815228%7CUnknown%7CTWFpbGZsb3d8eyJWIjoiMC4wLjAwMDAiLCJQIjoiV2luMzIiLCJBTiI6Ik1haWwiLCJXVCI6Mn0%3D%7C3000%7C%7C%7C&sdata=EJ5e8wI0uKTdCJVQQ1aLOHqQGnXnpf%2BwuX5TL4i7kqs%3D&reserved=0) requires authors to make all data underlying the findings described in their manuscript fully available without restriction, with rare exception. The data should be provided as part of the manuscript or its supporting information, or deposited to a public repository. For example, in addition to summary statistics, the data points behind means, medians and variance measures should be available. If there are restrictions on publicly sharing data—e.g. participant privacy or use of data from a third party—those must be specified.

Reviewer #1: Yes

Reviewer #2: Yes

4. Is the manuscript presented in an intelligible fashion and written in standard English?

PLOS Global Public Health does not copyedit accepted manuscripts, so the language in submitted articles must be clear, correct, and unambiguous. Any typographical or grammatical errors should be corrected at revision, so please note any specific errors here.

Reviewer #1: Yes

Reviewer #2: Yes

5. Review Comments to the Author

Please use the space provided to explain your answers to the questions above. You may also include additional comments for the author, including concerns about dual publication, research ethics, or publication ethics. (Please upload your review as an attachment if it exceeds 20,000 characters)

Reviewer #1: Review: Use of standardised patients to assess tuberculosis case management by pharmacies in Patna, India: a repeat cross-sectional study.

Overall
The manuscript is well written, technically sound and covers an important topic.
In my view the statistical analyses are appropriate and rigorous but further review by a statistician might be helpful.

The authors report that the datasets are available and there is a link in the manuscript where you can request for study tools. It is not clear to me how you obtain the datasets.

*The datasets will be made available at the time of publication at* [*https://www.qutubproject.org/*](https://www.qutubproject.org/) *and listed in a link with a link to the article and the de-identified dataset.*

Comments to the authors

With likely more 70% similarity to the preceding study, shouldn’t this manuscript have been published as update to the previous one?

*While the sampling may be similar from the previous study, there are some key differences between these papers, namely that almost all the analyses conducted are completely different from the first paper and that this paper sample solely from Patna. Therefore, it would not be an update but an entirely different study, that makes use of the baseline data available from a previous survey.*

Reading the manuscript, the information you want is all there but not where you would expect it for example:

1. Some key information like “what a standardised patient is” should be provided in the introduction as part of the summary of the previous study. As it is now, it leaves you guessing until much further in the manuscript.

*This is a very fair point. We have thus added the following to line 126 of Introduction: “In this current study, we again made use of standardized patients (SPs), a healthy individual trained to portray a clinical problem or situation for the purpose of better understanding specific behaviours.”*

2. Paragraph 463-469 should come after 437-446 then 513-524

*Absolutely agree. Have changed the order of the paragraphs for smoother readability.*

A bit more distinction between the 2 studies is needed maybe in a separate paragraph or table. The authors mentioned that 92% of the same pharmacies were used; were the SOPs, standardized patients, and study teams the same? If not what were the differences and what potential impact could these have had?

*We are not sure this is necessary given that the previous study was quite different in terms of content. The survey data used from this study and the sampling techniques were consistent, however, there are some key differences between these papers, namely that almost all the analyses conducted are completely different from the first paper and that this paper sample solely from Patna. Therefore, it would not be an update but an entirely different study, that makes use of the baseline data available from a previous survey.*

Lines 140-144… consider integrating this into the introduction section.

*Have changed the order/position of the Context paragraph.*

Lines 212 to 214, how many standardized patients were used in the second round? This doesn’t come out clearly.

*Agreed that this was not worded very well and has now been revised to the following: “In the 2014 round, SPs completed 500 visits to pharmacies in Patna. In the repeat survey, standardized patients were sent to 260 randomly sampled pharmacies in Patna between October 2019 and January 2020, resulting an additional 436 interactions (936 total interactions between baseline and second survey).”*

Line 217 PPIA should be defined here and not 224-225.

*Thank you for flagging this; have now defined PPIA on line 217 rather than 224-225.*

Figures: Please add headings and label all the axes.

*The figures are formatted as per the journal’s guidelines.*

The manuscript should be shortened e.g. by presenting only the key results and others left to an appendix.

*Since PLOS GPH journal does not have a word limit and we thought that all the analysis conducted would be of interest to readers, we included them all in the main body of the paper. This was done purposefully as the analyses are quite novel.*

In lines 526-545 the authors prescribe generic solutions and yet the study did not investigate the reasons for the decline in ideal and correct case management. This section should be removed or shortened because it is speculative.

*The wording of this section has been slightly altered to be less presumptive, but we do think it is important to provide readers with an understanding of what we authors believe the next step in investigation should be.*

Reviewer #2: Comments to the authors

Abstract and Introduction
Some sentences in the abstract are too long and confusing. The authors need to revise and cut short sentences for easy flow of the abstract: consider lines 44 to 48.

*We agree that the Abstract could use some work. We have therefore changed the wording of the first paragraph to the following: “As the first point of care for many healthcare seekers in resource-restrained settings, private pharmacies are positioned to play an important role in case detection and referral of tuberculosis (TB). However, previous studies in India have showed that private pharmacies commonly dispense cough syrups, bronchodilators, anti-histamines and broad-spectrum antibiotics over-the-counter (OTC), rather than referring patients to the appropriate provider for TB testing and treatment. Such inappropriate management by private pharmacies can contribute to delayed diagnosis and treatment for persons with TB.”*

The first and second sentences in the methods abstract section (lines 51-55) are more of study objectives, this can be pushed to the end of the introduction.

*In agreement with this; lines 51-55 have been moved to the abstract’s Introduction.*

The authors leave the reader hanging in their methods section of the abstract. A lot is missed out, issues on when the two surveys were conducted, sampling for the selected pharmacies, ought to come out clearly.

*We added in some specificity to the Methods section of the abstract in terms of temporality: “We examined how and whether private pharmacies improved practices for TB in 2019 compared to a baseline study conducted in 2015 in the city of Patna, using the same survey sampling techniques and principle study staff. The proportion of patient-pharmacist interactions that resulted in correct or ideal management, as well as the proportion of interactions resulting in antibiotic, quinolone, and corticosteroid are presented, with standard errors clustered at the provider level. To assess the difference in case management and the use of drugs across the two cases by round, a difference in difference (DiD) model was employed.”*

Generally, the introduction is well written, flows well and illustrates the gap and study problem.

The terms “private pharmacies” and “pharmacies” are interchangeably used throughout the paper. The authors need to use one term for consistence, remember public pharmacies also exist. For example, consider introduction line 99 to 105.

*This is an excellent point and a clear oversight in precision of language. The generic term “pharmacy” has been replaced by “private pharmacy” throughout the entirety of the paper.*

What does the term “interaction” mean in the paper? Is this patient-pharmacist interaction? It is not very clear and leaves the reader wondering at first read.

*Completely fair point; have altered the wording in the Introduction to be “patient-pharmacist interaction (consultation)” so that the reader does not confuse this with a statistical interaction.*

Is it okay to use the term standardized participant for patients presenting with classic symptoms of pulmonary TB? Is this a standard thing? It is not clear who a standardized patient is at first read in the introduction

*A similar point was brought up by Reviewer #1; the term standardized patient has now been defined in the Introduction to reflect this confusion.*

Line 86, there is a repetition of the word “In”, revise this

*Thank you for flagging! This has now been changed.*

Line 86 to 89, there is poor consistence and coherence between the sentences, consider revising the joining sentence of “These people, considered ‘missing’, are either undiagnosed or not notified to the TB programme, and comprise around 4.2 million cases…”. One wonders where these people come from; feels like they are already talked about yet they are not yet.

*Agreed. The wording has now been changed to: “These “missing” patients, individuals who are either undiagnosed or not notified to the TB programme, are estimated to amount to 4.2 million cases yearly. Failing to identify these persons results in continued community transmission, undermining national and global TB control efforts.”*

Methods
The methods are well detailed and explained fully.

Now the term “interactions” clearly comes out in the methods. It would be better to rephrase it to patient-pharmacist interaction the first time it appears in the paper for the reader to easily follow.

*Appreciate this suggestion immensely as it certainly provides greater clarity to the reader. This same point was addressed as per Reviewer #1’s similar guidance.*

Table 1 can be pushed to supplementary materials

*Respectfully, we believe Table 1 is essential for a clear understanding of the two case presentations. PLOS GPH has no word limit and it is more useful for readers to see the table in the main paper rather than supplement.*

Line 213, it is not clear how the authors come up with the fixed sample size of 805 additional interactions.

*Absolutely fair point and thank you for flagging, as Reviewer #1 had done. The wording has now been changed for greater clarity to: “In the 2014 round, SPs completed 500 visits to pharmacies in Patna. In the repeat survey, standardized patients were sent to 260 randomly sampled pharmacies in Patna between October 2019 and January 2020, resulting an additional 436 interactions (936 total interactions between baseline and second survey).”*

Definition of the outcome: lines 254-258: How well did you obtain information regarding the verbal referral, what was the proportion of those who had received the verbal referral; is this reliable anyway?

*Our staff of standardized patients was very well-trained to debrief their interactions to study management teams; so, while we cannot give you an exact break down of written vs verbal referral as this distinction was not captured in the data, we are quite confident we can reliably trust the SPs we trained for accuracy.*

Statistical analysis: Line 261-262; The sentence is in future tense, yet this was already done, please revise it.

*Appreciate the flag in grammar. Line 261-262 have been changed to the appropriate tense.*

Results
Results are well presented and explained; good work done.

Discussion and conclusion
Line 437 to 448; second paragraph is doing a lot of describing results which was already done in the results section; authors need to summarize the results here and compare and contrast their results with previous studies in similar contexts and give implications for their results.

*This was a stylistic choice that we would like to stand by. We agree that the paper is quite long, with numerous findings and several complex analyses. We thought it would be important to ground the reader in the main and most interesting findings in order to position them well before diving into the discussion.*

Line 463 to 469; While the authors mention the decline in progress between rounds of data collection by case and give it’s implications, they do not explain the possible explanation for the decline amidst the interventions done in the country. Adding a possible explanation adds value to this.

*This was detailed further on in the Discussion section, taking into account the various other findings (Line 670-690)*

General comment: This is a good paper that adds value to the TB knowledge and pharmacy prescription practices.

*Very kind! Thank you!*

6. PLOS authors have the option to publish the peer review history of their article ([what does this mean?](https://can01.safelinks.protection.outlook.com/?url=https%3A%2F%2Fjournals.plos.org%2Fglobalpublichealth%2Fs%2Feditorial-and-peer-review-process%23loc-peer-review-history&data=05%7C01%7Canita.svadzian%40mail.mcgill.ca%7C9ecd4c3930ad41c7bada08db2aa55fb8%7Ccd31967152e74a68afa9fcf8f89f09ea%7C0%7C0%7C638150661979815228%7CUnknown%7CTWFpbGZsb3d8eyJWIjoiMC4wLjAwMDAiLCJQIjoiV2luMzIiLCJBTiI6Ik1haWwiLCJXVCI6Mn0%3D%7C3000%7C%7C%7C&sdata=T%2BPE4RSe2KXZd69XkhrLYqkJwhRLz3sMo29ldFDb%2Br4%3D&reserved=0)). If published, this will include your full peer review and any attached files.


**Do you want your identity to be public for this peer review?** If you choose “no”, your identity will remain anonymous but your review may still be made public.


For information about this choice, including consent withdrawal, please see our [Privacy Policy](https://can01.safelinks.protection.outlook.com/?url=https%3A%2F%2Fwww.plos.org%2Fprivacy-policy&data=05%7C01%7Canita.svadzian%40mail.mcgill.ca%7C9ecd4c3930ad41c7bada08db2aa55fb8%7Ccd31967152e74a68afa9fcf8f89f09ea%7C0%7C0%7C638150661979815228%7CUnknown%7CTWFpbGZsb3d8eyJWIjoiMC4wLjAwMDAiLCJQIjoiV2luMzIiLCJBTiI6Ik1haWwiLCJXVCI6Mn0%3D%7C3000%7C%7C%7C&sdata=QJzrex52%2BirYIrx8F%2Fob2PobyIUivaMIavTTwggefTk%3D&reserved=0).

Reviewer #1: No

Reviewer #2: No
